# Perceptual Learning of Motion Direction Discrimination with Suppressed and Unsuppressed MT in Humans: An fMRI Study

**DOI:** 10.1371/journal.pone.0053458

**Published:** 2013-01-09

**Authors:** Benjamin Thompson, Bosco S. Tjan, Zili Liu

**Affiliations:** 1 Department of Optometry and Vision Science, University of Auckland, Auckland, New Zealand; 2 Department of Psychology and Neuroscience Graduate Program, University of Sothern California, Los Angeles, California, United States of America; 3 Department of Psychology, University of California, Los Angeles, California, United States of America; University of Sydney, Australia

## Abstract

The middle temporal area of the extrastriate visual cortex (area MT) is integral to motion perception and is thought to play a key role in the perceptual learning of motion tasks. We have previously found, however, that perceptual learning of a motion discrimination task is possible even when the training stimulus contains locally balanced, motion opponent signals that putatively suppress the response of MT. Assuming at least partial suppression of MT, possible explanations for this learning are that 1) training made MT more responsive by reducing motion opponency, 2) MT remained suppressed and alternative visual areas such as V1 enabled learning and/or 3) suppression of MT increased with training, possibly to reduce noise. Here we used fMRI to test these possibilities. We first confirmed that the motion opponent stimulus did indeed suppress the BOLD response within hMT+ compared to an almost identical stimulus without locally balanced motion signals. We then trained participants on motion opponent or non-opponent stimuli. Training with the motion opponent stimulus reduced the BOLD response within hMT+ and greater reductions in BOLD response were correlated with greater amounts of learning. The opposite relationship between BOLD and behaviour was found at V1 for the group trained on the motion-opponent stimulus and at both V1 and hMT+ for the group trained on the non-opponent motion stimulus. As the average response of many cells within MT to motion opponent stimuli is the same as their response to non-directional flickering noise, the reduced activation of hMT+ after training may reflect noise reduction.

## Introduction

Perceptual learning is an established example of neural plasticity within the adult brain whereby practice of a perceptual task leads to a pronounced improvement in behavioural performance [1]–[3]. The behavioural effects of perceptual learning have been widely studied within the visual system and early investigations found that the learning tended to be highly specific to the trained stimulus [4]–[7]. This led to the suggestion that improvements in task performance may result from changes at an early stage of visual processing where neurons encode highly specific visual features. The presence of changes at an early stage of visual processing has been supported by a number of human neuroimaging studies [8]–[15] as well as neurophysiological investigations of perceptual learning in animals [16]–[24]. However, the low-level changes found in a number of neurophysiological studies have not been sufficient to explain the full extent of the behavioural improvements that resulted from perceptual learning [19], [20], [23], [25], [26] and plasticity in higher-level decision making areas has been reported [26]. On balance, the current evidence suggests that perceptual learning may reflect changes in early visual brain areas, brain areas involved in decision making, or both, depending on the task and the nature of the training [27].

In the case of motion tasks, perceptual learning is thought to involve the middle temporal area (MT), a region strongly implicated in the processing of motion information in both primates [28]–[30] and humans [31]. An early, influential neurophysiological investigation [32] found that when monkeys were trained to discriminate the direction of coherent motion embedded in noise over a block of 400 trials, improvements in accuracy were well correlated with increases in the directional sensitivity of neurons within MT and the middle superior temporal area (MST). The only fMRI study to investigate motion perceptual learning published to date reported an increase in the BOLD response within a cortical area corresponding to hMT+ (the human homologue of MT and MST). This increase in response occurred during the rapid acquisition of a motion coherence task in naïve participants during a single scanning session [33]. It is not clear, therefore, whether this effect reflected long term, low-level plasticity or more general processes involved in task acquisition.

These early studies have recently been extended by an investigation into the effects of prolonged motion coherence training in monkeys on the response of single cells within MT and the higher-level lateral interparietal area (LIP). Law and Gold (2008) found that activity within MT became more predictive of behavioural accuracy as a function of training and that the sensitivity of single cells increased during individual training sessions. However, stronger relationships were found between changes in the activity of neurons within LIP and behavioural improvement, suggesting that the learning resulted from an increasingly accurate read-out of largely unchanged MT signals.

In general therefore, the current human neuroimaging and primate neurophysiological evidence indicates that information carried by MT is central to perceptual learning of motion tasks, even though MT may not be the locus of long term plasticity. This is consistent with the finding that lesions of MT greatly impair the ability of monkeys to improve at a motion coherence task despite extensive training [34]. A partially analogous effect has been shown psychophysically in humans using a stimulus that employs motion opponency to suppress the response of MT to task relevant motion information [35]. Motion opponency refers to a property of some directionally selective neurons in MT that are inhibited by motion in their anti-preferred directions [36], [37]. For example, Qian & Andersen [36] found that the response of MT was suppressed when a motion signal was carried by pairs of dots that moved in counter-phase to one another, thereby nulling the local and global motion directions within the stimulus. Importantly, Qian & Andersen [36] showed that the average response of MT neurons to the paired-dot stimulus was no greater than the response to flickering noise. Evidence for motion opponency has also been found in fMRI studies of human hMT+ using counterphase gratings [38], [39] and paired vs. unpaired dots [38]. Motion opponent interactions may also explain the reduced BOLD response found at MT and MST for coherent relative to incoherent motion of plaid stimuli [40].

**Figure 1:**
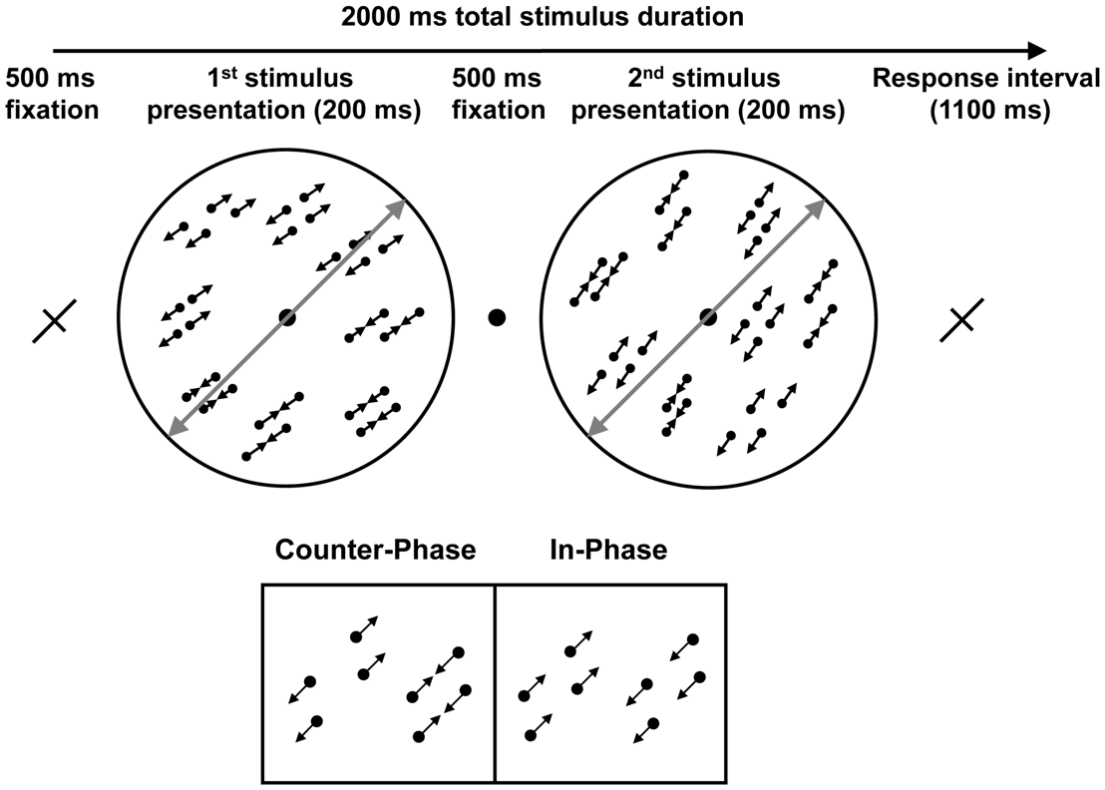
A schematic representation of a single trial demonstrating a counter-clockwise change in motion axis about a bisecting motion axis of 45°. The bisecting axis is depicted with solid grey lines (not present in the real stimuli). The lower panel illustrates the two possible relative phases of dot motion. Counter-phase dots provided locally balanced motion directions and therefore putatively suppressed MT. In-phase dots did not have locally balanced motion signals and therefore MT was presumed to be activated normally. Arrows are shown for illustrative purposes only.

Lu et al. [35] modified Qian & Andersen’s [36] original stimulus to allow for a motion axis discrimination task to be performed. In addition, rather than using paired vs. unpaired dots to control the presence or absence of motion opponency, Lu et al. [35] simply reversed the phase of the paired dot motion to remove motion opponency while leaving all other aspects of the stimuli and task unchanged. Specifically, in the motion opponent stimulus, the two dots in a pair moved towards and away from one another (counter-phase motion) whereas in the non-opponent motion stimulus the dots in a pair moved back and forth in unison (in-phase motion). Therefore in-phase motion preserved the global balancing of motion signals in the stimulus, but destroyed the precise local balancing necessary for motion opponency [36] (Figure 1).

Lu et al. (2004) found that when the task required fine differences in motion axis to be resolved, no perceptual learning was possible even though behavioural accuracy was above chance. However, when task difficulty was relaxed, learning was enabled. This result was replicated by Thompson and Liu [41], who also showed that perceptual learning was equivalent for both counter-phase and in-phase dots when the task required relatively coarse judgements of motion axis orientation.

These results raised an interesting question: what is the nature of the learning that takes place for the counter-phase dot stimulus? One possibility is that perceptual learning causes a reduction in motion-opponency at MT, allowing for a stronger signal. Another possibility is that the response of MT remains suppressed or becomes even more suppressed to prevent the motion-opponency subsystem from adding noise to the rest of the system. In this case perceptual learning could rely on the subset of neurons within MT that do not exhibit motion opponency. Alternatively, other visual brain areas that are sensitive to motion direction such as V1 may be recruited. In the present study we used fMRI to measure the BOLD response at hMT+ and V1 before and after learning to investigate these possibilities. Two groups of participants were trained extensively using either counter-phase or in-phase dots at a task difficulty that was permissive for learning [41]. Training on counter-phase dots resulted in a training-specific reduction in BOLD response at hMT+ and greater reductions were correlated with greater amounts of learning. The opposite relationship between learning and BOLD was present at V1 for the counter-phase group and at both V1 and hMT+ for the in-phase group. One possible explanation for these results is that training on the counter-phase dots led to reduced noise within the neural signal carried by MT.

## Methods

### Ethics Statement

All study protocols were approved by the University of California Los Angeles and the University of Southern California institutional ethical review boards and were in agreement with the Declaration of Helsinki.

### Participants

Twenty participants (age range 20–34 years, 8 females) provided written, informed consent and took part in the study. Eleven participants were trained on counter-phase dot stimuli, seven were trained on in-phase dot stimuli and two took part in the pre training stimulus-validation scans only.

### Stimuli and Task

The task was to discriminate a clockwise or counter-clockwise change in motion axis defined by the common trajectories of a field of moving dots. Dots were grouped into pairs within which they moved either in-phase or counter-phase (Figure 1). Pairs of dots within the counter-phase stimulus oscillated back and forth in opposite directions and were presumed to suppress MT [36], [38], whereas in-phase paired dots oscillated together and provided a strong directional motion signal. To balance the average motion direction for the in-phase dot stimulus, at any moment, half of the dots in the display moved in one direction and other half moved in the opposite direction. The stimuli and task have been described previously [35], [41] and were modified from a stimulus developed by Qian & Andersen [36].

Each stimulus consisted of a grey circular aperture containing 100 dot pairs. Dot pairs were further arranged into “twin-pairs”, a manipulation that destroyed the Glass pattern [42] so that no orientation information was present in any static frame of the display [35]. The orientation along which the dots oscillated was kept constant over pairs so that each stimulus had a global motion orientation (termed motion-axis). Within a single trial, two stimuli were presented sequentially and participants judged the direction of the motion-axis change within a 1100 ms response window. The stimulus duration was 200 ms and a 500 ms inter-stimulus-interval was included to prevent apparent motion cues from one stimulus to the next. The average motion axis across the two stimuli within a trial was constant at either 45° or 135°. This bisecting motion axis was indicated by the long arm of a fixation cross that was presented before and after each trial (Figure 1).

Stimuli were generated and presented using Matlab and the psychophysics toolbox [43], [44]. The stimulus aperture subtended 7.8° of visual angle and each dot subtended 0.06° of visual angle. The minimum distance between two dots in a pair was 0.06° (dots were not allowed to overlap) and the maximum was 0.3°. The two pairs making up a twin-pair were positioned 0.06° to 0.15° apart from one another to form a parallelogram. Dots moved at a speed of 2°/sec and had a lifetime of 120 ms. When one twin pair disappeared, a new twin pair appeared at a random location within the display aperture. The lifetime of twin pairs was randomized using a flat distribution of 60 ms ±17 ms. The starting inter-dot distance for the counter-phase dot pairs was randomized using a flat distribution of 0.15° ±0.03°. Twenty percent of twin pairs were each assigned a random motion axis and acted as noise to encourage participants to view the entire display. If a participant’s discrimination threshold at 75% correct was less than 8°, the noise density was increased. In the laboratory, stimuli were presented in a darkened room on an NEC MultiSync FE771SB monitor with a vertical refresh rate of 60 Hz and a resolution of 800×600 pixels. A chin-rest maintained a constant viewing distance of 120 cm, which was also the length of a dark viewing tube that abutted the monitor to reduce extraneous orientation cues. Participants performed the discrimination task using two keys on a computer keyboard. The laboratory viewing conditions were designed to closely mimic the appearance of the stimuli through the VisuaStim XGA fMRI compatible viewing goggles which were used during scanning. The goggles had a resolution of 800×600 pixels and a 60 Hz vertical refresh rate. An MRI compatible button box was used to collect behavioural responses during scanning.

### Procedure

#### Perceptual learning

Participants practiced the task with a 30° difference in motion axis and trial-wise auditory feedback until 95% correct accuracy was achieved for both the 45° and 135° bisecting motion axes over a block of 100 trials. After practice, psychometric functions were measured for each participant. Accuracy was assessed for 4°, 8°, 12°, 16°, and 20° differences in motion axis. Each level was presented within a blocked design without feedback. There were 40 trials per block and each block was presented twice. Block order was randomized for the first presentation and counter balanced for the second presentation. Each participant completed at least two psychometric curve measurements for each bisecting motion axis (45° and 135°). 75% correct thresholds were calculated by fitting a Weibul function to the final curve measurements. The average threshold across the 45° and 135° axes was used to set task difficulty for the pre-training fMRI scan and for the first session of training. Initial measurements were made with 20% of the twin pairs presented as noise. If threshold values were 8° or less, curves were re-measured with increased noise densities until a higher angular threshold was reached to ensure that there was room for learning to occur. Prior to scanning a short psychometric function (20 trials per point) was measured for each bisecting motion axis inside the MRI scanner bore to ensure that the laboratory measurements transferred to the scanner environment.

During training, behavioural accuracy was fixed at 75% correct and task difficulty was manipulated by varying the angular difference in motion axis. Participants were trained with feedback in daily blocks of 400 trials along a bisecting motion axis of either 45° or 135° (randomized across participants). Task difficulty was kept constant during a block and if 75% accuracy or better was achieved, the angular size for the subsequent training session was reduced by 1°. If accuracy was less than 75% then task difficulty was kept constant for the next training session. Training continued until the initial angular size had been halved or learning reached an asymptote. After training, psychometric functions were re-measured along each motion axis to define the angular size for use in the post training scanning session.

#### fMRI

For all but three participants scanning was conducted using a 3T Siemens Allegra MRI scanner. Each session began with the acquisition of medium resolution T1-weighted 3D anatomical images (20 saggital slices 0.8×0.8×4 mm^3^, TR 4700 ms, TE 56 ms) that were used to guide the prescription of slices for functional imaging. Functional measurements (echo-planar images, TR = 2500 ms, TE = 50 ms, 90° flip angle) consisted of 29 slices (3.1×3.1×4 mm) oriented perpendicular to the calcarine sulcus which covered the whole occipital lobe. A high-resolution anatomical image of the whole brain was also acquired using a magnetization prepared rapid gradient echo (MPRAGE) sequence (TR = 2300 ms, TE = 2.11 ms, Flip angle = 8°, TI = 1100 ms). The remaining three participants completed all scans on a 3T Siemens Tim Trio MRI scanner with a slightly different functional protocol (EPI, 32 slices, 3×3×3 mm, TR = 2000 ms, TE = 30 ms, 90° flip angle).

**Figure 2:**
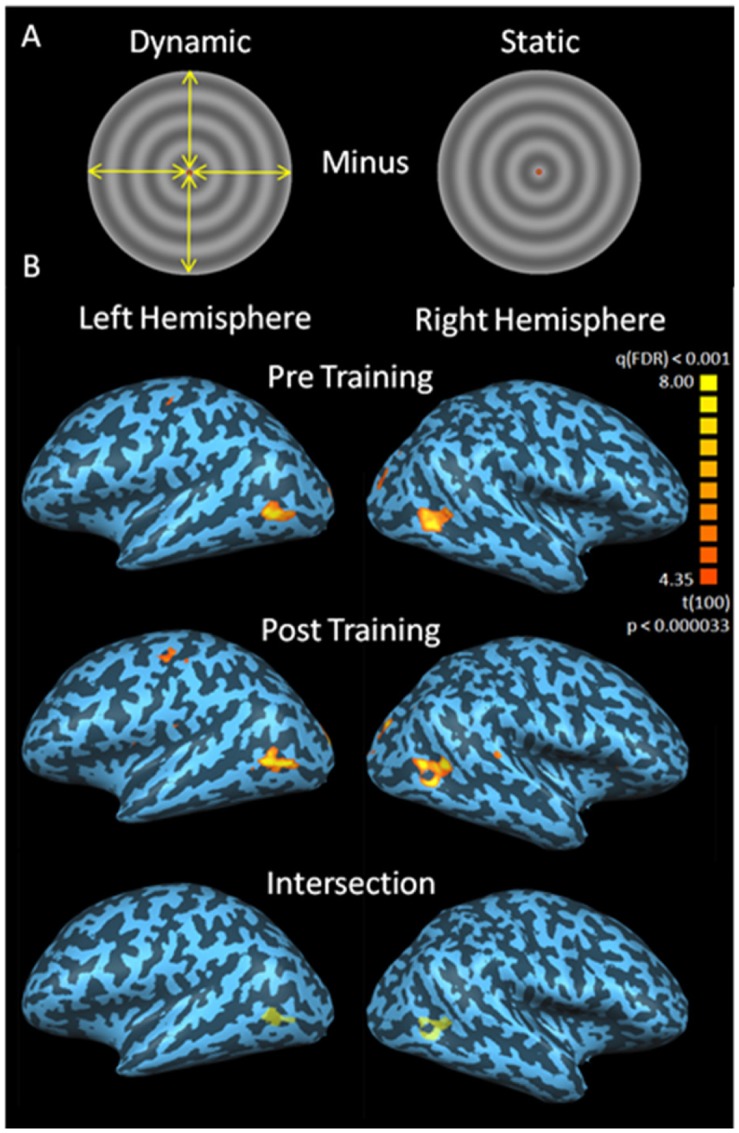
Localization of hMT+. The hMT+ localization stimulus is shown in panel A with yellow arrows representing the centripetal oscillations that occurred during the dynamic phase. Example localization results for one participant are shown in panel B. hMT+ localization data were acquired pre (top row) and post (middle row) training. The hMT+ ROI used for analysis was derived from the intersection of the pre and post training ROIs (bottom row). The FDR corrected (q<0.001) statistical maps are rendered on inflated representations of the participant’s left and right cerebral hemispheres.

Within the pre-training scanning session, participants completed a minimum of four functional scans during which they performed the motion axis discrimination task at 75% correct threshold. Four conditions (45° and 135° bisecting motion axes, counter-phase and in-phase dots) were presented within each scan for the group to be trained on counter-phase dots and the two participants who did not complete training. For the group to be trained on in-phase dots, only in-phase dots (45° and 135° motion axes) were presented. A blocked design was employed whereby each condition was presented twice for the counter-phase dot participants and four times for the in-phase dot participants. Motion axis, angular size, and dot phase were kept constant during a block. Each block contained 10 trials, lasted 20 seconds, and consecutive blocks were separated by 20 seconds of mean luminance fixation. The order of the blocks was randomized for the first four blocks and counter-balanced for the last four. Following the motion axis discrimination scans, retinotopic mapping data were collected using standard wedge and ring stimuli [45] (maximum diameter = 16° of visual angle) to allow for localization of V1. Finally, participants completed an hMT+ localization scan during which they viewed alternating 20 second blocks of dynamic and static radial sinusoidal grating stimuli identical in size to the motion axis discrimination stimuli (7.8° diameter) (Figure 2). During the six dynamic blocks, the radial grating (0.4 cpd) expanded and contracted at 9 Hz with the motion direction changing every 2.5 seconds to avoid the induction of a motion aftereffect. Participants were required to fixate centrally and press a button every time the grating changed direction, started or stopped.

The post training scans included a minimum of four functional scans during which participants completed the motion axis discrimination task. Each scan contained four conditions repeated twice in a randomized, counterbalanced manner. For the participants trained on in-phase dots, in-phase stimuli were presented along the trained and untrained bisecting motion axis at two difficulty levels. The first difficulty level used stimuli identical to those presented during the pre-training scans for both the trained and untrained bisecting motion axis. The second difficulty level was set at the 75% correct threshold measured after training for each bisecting motion axis. This accounted for training related improvement along the trained axis and transfer of learning to the untrained axis. These two difficulty levels are referred to as the “same angle” and “smaller angle” conditions respectively. Nine of the participants trained on the counter-phase dot stimuli completed an identical scanning sequence with only counter-phase dot stimuli presented. In addition, eight participants who were trained on the counter-phase dots viewed a different set of conditions post training. These participants were presented with both in-phase and counter-phase dots post training at the difficulty level that corresponded to 75% correct post training (i.e., the “smaller angle” condition). Six participants trained on the counter-phase dots completed both post-training scan protocols. All post training scans also included an hMT+ localization sequence. Table 1 outlines the stimuli presented during the pre and post training scans.

**Table 1:** An outline of the experimental design**.**

	In-phase dot training	Counter-phase dot training (total n = 11; n = 6 completed both protocols)	
	Protocol 1 (n = 7)	Protocol 1 (n = 9)	Protocol 2 (n = 8)
Pre-training scan: stimuli	In-phase dots only	In-phase & counter-phase dots	In-phase & counter-phase dots
Pre-training scan: task difficulty	Same angle only	Same angle only	Same angle only
Post-training scan: stimuli	In-phase dots only	Counter-phase dots only	In-phase & counter-phase dots
Post-training scan: task difficulty	Same and smaller angle	Same and smaller angle	Smaller angle only

The main experiment employed protocol 1 whereby learning related changes in the response of hMT+ and V1 were compared between two groups, one trained on in-phase dots and the other trained on counter-phase dots. Protocol 2 applied only to the group trained on counter-phase dots and compared the response of hMT+ and V1 to in-phase vs. counter-phase dots pre and post training (within subjects). A total of 11 participants were trained on counter-phase dots and 6 completed both scanning protocols after training. Same angle refers to the angular difference in motion axis orientation that gave rise to 75% correct before training. Smaller angle refers to the angular difference corresponding to 75% correct after training.

fMRI data were analysed using BrainVoyager. Functional data were motion corrected, high-pass filtered and registered to Talairach space using sub-routines within BrainVoyager. Polar maps were analysed on a flattened representation of the occipital lobe and V1 was defined as a cortical region flanked by polar angle phase reversals and bisected by the calcarine sulcus. To ensure that only stimulus responsive voxels were included within the V1 region of interest (ROI), a single subject general linear model analysis was conducted for each participant. Voxels within V1 that were activated above a false discovery rate of q<0.001 for a contrast between all stimulus presentation blocks and all blank fixation blocks before or after training were included in the bilateral V1 ROI. Area hMT+ was defined as a region of contiguous voxels in the correct anatomical region [46] that showed greater activation to dynamic than static radial grating stimuli after an FDR correction of q<0.001. hMT+ was defined separately pre and post training for each participant and only voxels that were activated both pre and post training (q<0.001) were included in the hMT+ ROI (Figure 2).

Measures of the BOLD response were calculated by extracting the raw time series data from the V1 and hMT+ ROIs and averaging these data across the voxels within each ROI. This was done for each scanning run for each participant. These raw data were converted to units of percent signal change by normalizing to the last two TRs of the directly preceding blank fixation block. An average %BOLD change was then calculated by averaging the values of individual time points that fell within a time widow that was the same duration as the stimulus presentation (20 seconds, 8 TRs) but shifted by 5 seconds (2 TRs) to account for the hemodynamic delay.

## Results

### Behavioural Effects of Perceptual Learning

**Figure 3:**
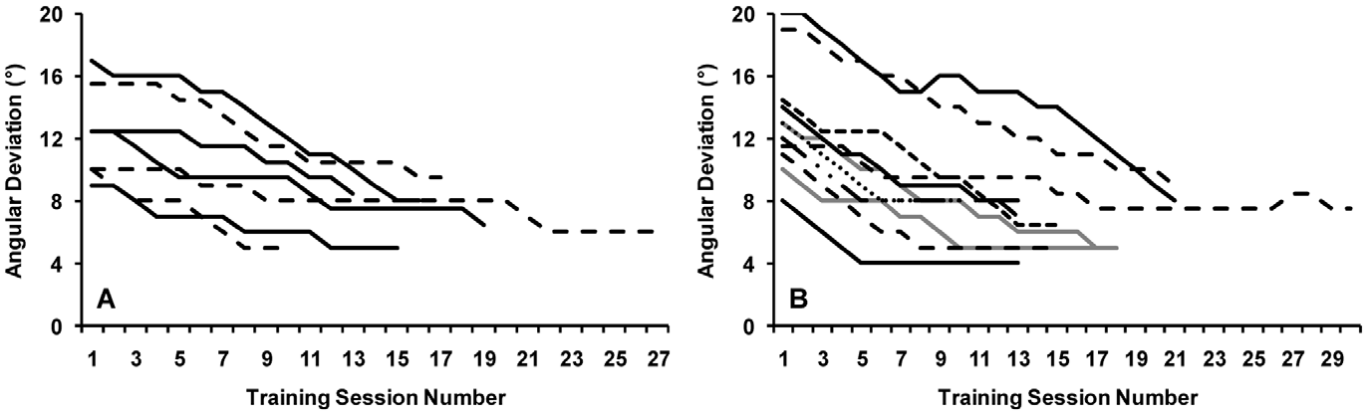
Improvement in behavioural thresholds as a result of learning for participants trained on in-phase dots (A) and counter-phase dots (B). Each training session consisted of 400 trials. If 75% correct or better was achieved during a training session, the angular deviation was decreased by 1° for the subsequent training session. For two participants trained on counter-phase dots, the angular deviation was increased for two sessions mid-training in an attempt to facilitate additional learning. Each line represents an individual participant.

**Figure 4:**
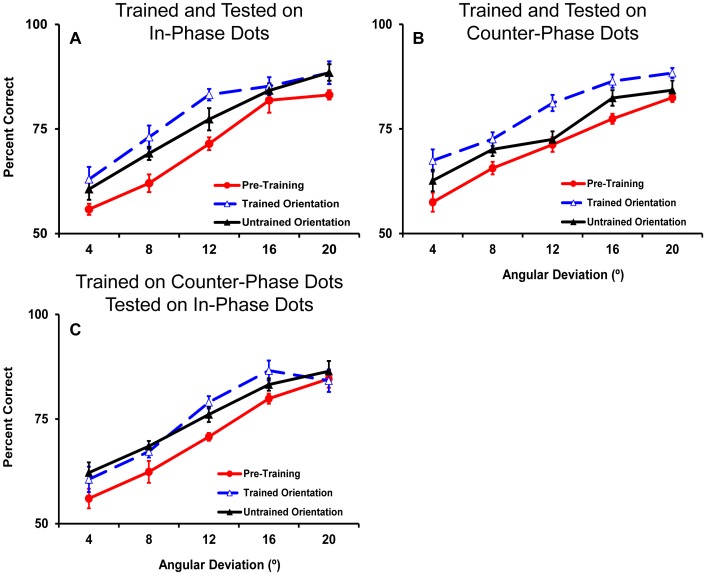
Average pre and post training psychometric functions for participants trained on in-phase dots (n = 7) (A) and counter- phase dots (B) (n = 11). A subset of 5 participants trained on counter- phase dots also completed psychometric functions for in- phase dots pre and post training. These data are shown in panel C. For each set of functions pre training data are collapsed across the two motion axis orientations as they did not differ. Error bars show within subjects standard error of the mean [53], [54] in this figure and all subsequent figures that represent within subject effects.

Training significantly reduced the motion axis orientation difference required for 75% accuracy (Figure 3 and Figure 4) and this improvement was more pronounced for the trained than the untrained motion axis. For the group of 7 participants trained on the in-phase dots, the threshold angular difference for the trained motion axis was reduced from a pre training average of 12°±3° to 7°±2° (t_6_ = 7.1, p<0.001). There was also a significant transfer of learning to the untrained axis for which the threshold was reduced from 12°±3° to 9°±3° (t_6_ = 4.1, p = 0.006). There was significantly more learning for the trained (43%±10% mean improvement) than the untrained (27%±13% mean improvement) motion axis (t_6_ = 3.5, p = 0.01).

For the group of 11 participants trained on the counter-phase dots, training significantly improved the threshold for 75% correct accuracy from a pre training average of 13°±4 to 7°±1° (t_10_ = 7.4, p<0.001) for the trained motion axis. Significant transfer of learning to the untrained motion axis also occurred for this group with thresholds dropping from 13°±4° pre training to 10°±4° post training (t_10_ = 2.5, p = 0.03). Again there was significantly more learning for the trained (46%±9% mean improvement) than the untrained (21%±22% mean improvement) motion axis (t_10_ = 3.3, p = 0.008). Importantly, the group trained on in-phase dots and the group trained on counter-phase dots did not differ in the amount of learning (F_1,16_<1) or transfer (improvement for the untrained motion axis, F_1,16_<1) that resulted from training.

Of the 11 participants trained on the counter-phase dots, 5 were also tested psychophysically with in-phase dots both pre and post training. Consistent with the previous results [35], training on counter-phase dots transferred to the in-phase dot stimuli with thresholds reducing from 14°±4° pre training to 10°±4° post training (t_4_ = 3.5, p = 0.02) for the trained motion axis orientation. A comparable amount of transfer also occurred for the untrained motion axis with thresholds changing from 14°±4° pre training to 10.5°±4° post training (t_4_ = 2.6, p = 0.06). The improvement for the trained orientation (27%±16%) did not reliably differ from the improvement for the untrained orientation (24%±22%). In addition, the improvement along the trained orientation was only marginally less than that exhibited by the group trained on in-phase dots (t_10_ = 2.1, p = 0.06). Pre and post training psychometric functions are shown for all conditions in Figure 4.

### Functional Magnetic Resonance Imaging

#### Pre training motion opponency

**Figure 5:**
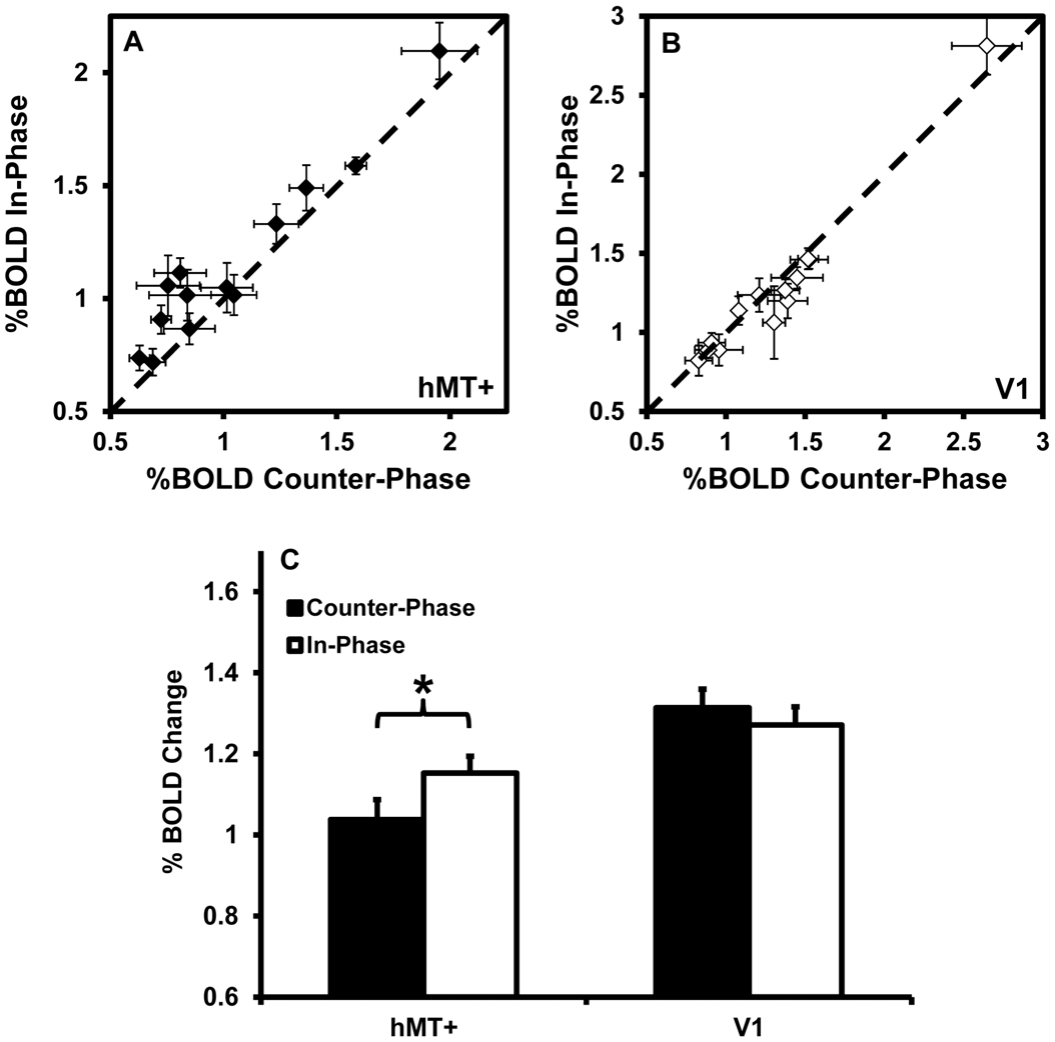
The response of hMT+ and V1 to counter-phase vs. in-phase dot stimuli before training (n = 13). Data for individual participants are shown in panel A for hMT+ and panel B for V1. Data points lying above the unity line indicate a greater response to in-phase dots, consistent with motion opponency. Group averages are shown in panel C. * indicates a statistically significant difference (p<0.01).

To test the hypothesis that counter-phase dot motion would suppress MT activity [36]–[38], we compared the BOLD response of hMT+ and V1 to counter-phase and in-phase dot motion prior to training. A within-subjects ANOVA with factors of visual area (hMT+ vs. V1), motion axis (45° vs. 135°) and dot phase (counter-phase vs. in-phase) revealed a significant interaction between visual area and dot phase, F_1,12_ = 14.0, p = 0.003, indicating that hMT+ and V1 responded differently to dot phase. As there was no effect of motion axis (F<1), data were collapsed across this variable for post-hoc analysis. hMT+ showed a significantly greater response to the in-phase dots than to the counter-phase dots, t_12_ = 3.9, p = 0.002 (Figure 5). There was no effect of dot phase on the response of V1, t_12_ = 1.4, p = 0.2. These results support the idea that counter-phase dots suppress the response of hMT+, presumably due to motion oponency [36], [38]. This effect could not be attributed to differences in task difficulty, as behavioural accuracy did not differ between the counter-phase and in-phase stimuli during scanning (mean accuracy was 76.5% correct for both stimuli, p>0.9). This experiment differs from the study performed by Heeger et al. [38] as we compared counter-phase with in-phase paired-dots, whereas Heeger et al. compared counter-phase paired-dots with unpaired dots.

#### Training on counter-phase dots vs. in-phase dots

**Figure 6:**
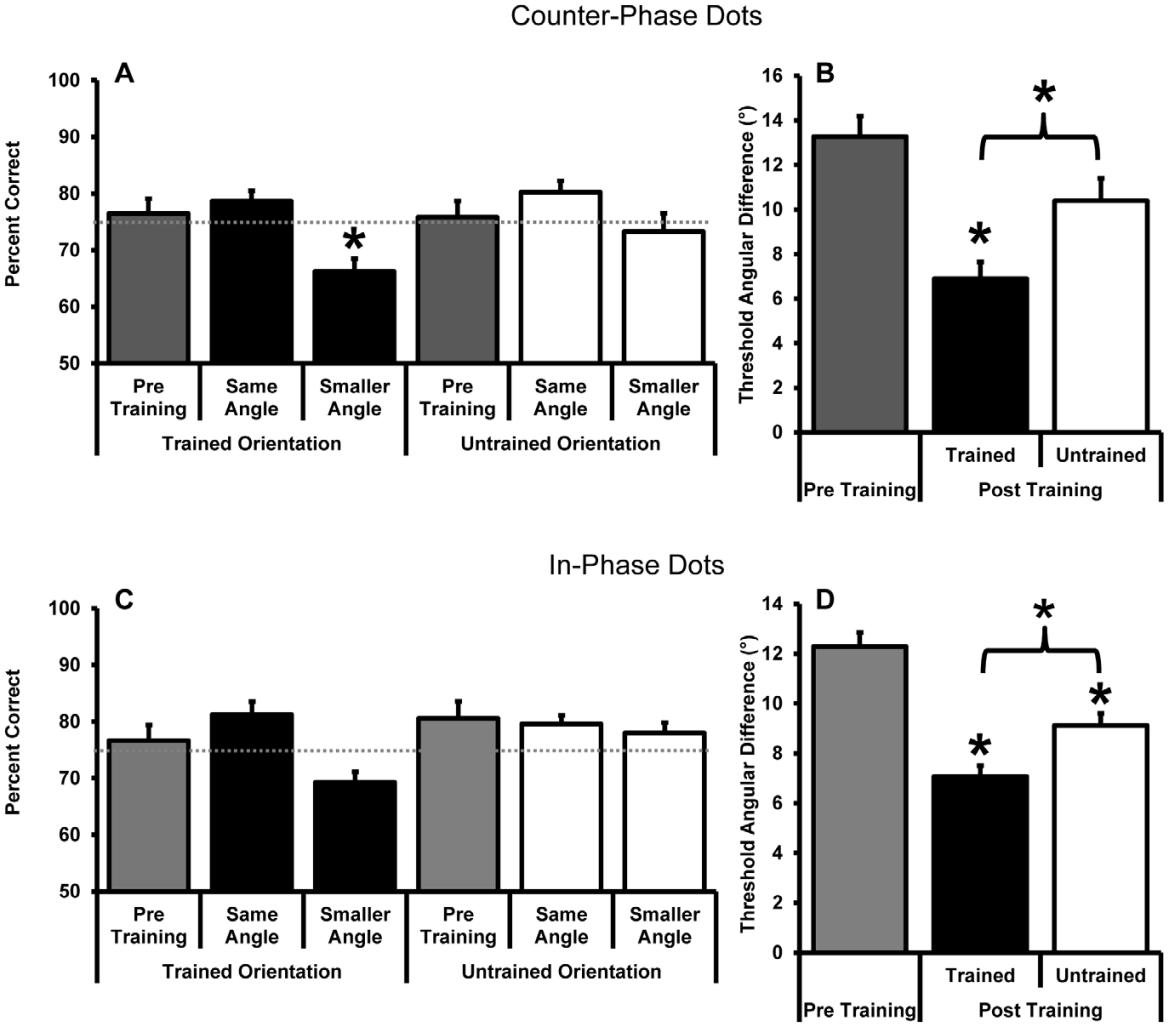
Behavioural accuracy during scanning (A and C) and the average angular threshold that gave rise to 75% correct accuracy outside of the magnet (B and D) for the group of participants trained on counter-phase dot stimuli (n = 9) (A and B) and those trained on in-phase dot stimuli (n = 7) (C and D). Data are shown for measurements made pre and post training. Post training stimuli were presented either at the same angular size shown pre training (“same angles” in panels A and C which correspond to pre-training measurements in panels B and D) or at the 75% correct angular threshold as measured outside of the magnet post training (“smaller angles” in A and C which correspond to the post training measurements in panels B and D). The dashed grey lines in A and C indicate 75% correct behavioural accuracy. Asterisks indicate a significant difference from pre training data unless otherwise indicated by a bracket (paired samples t-tests, p<0.05).

Next we investigated whether learning-related changes occurred at hMT+ and V1 for participants trained on either the counter-phase (n = 9) or in-phase (n = 7) dot motion stimuli. Participants were scanned at two angular differences post training; a “same angle” which was the same as the angle used during the pre-training scan and a “smaller angle” that gave rise to 75% accuracy after training. The smaller angle was calculated separately for the trained and untrained motion axes to account for learning and transfer respectively. After training, the angular difference that gave rise to 75% accuracy in the laboratory gave rise to a lower accuracy in the magnet; however, accuracy remained well above chance (Figure 6) and did not differ between the counter-phase and the in-phase training groups. This indicated that although the pre-training threshold transferred from the laboratory to the magnet, the learning was partially specific to the training environment. Similar issues have been reported previously [12].

**Figure 7:**
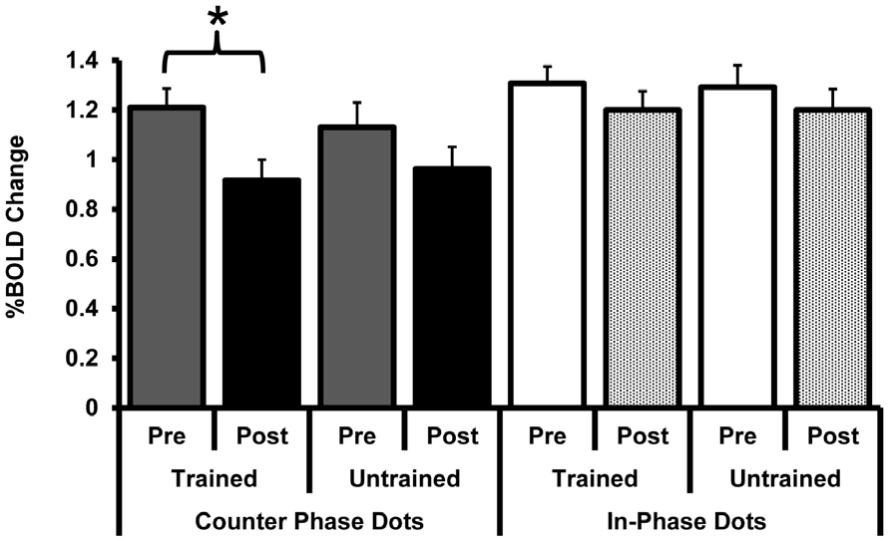
The response of hMT+ (%BOLD change) pre and post training with counter-phase dots (filled bars) or in-phase dots (open/textured bars). * indicates a significant reduction in the activation of hMT+ for the counter-phase dot stimulus presented along the trained motion-axis. Post training data are collapsed across angle size (“same” and “smaller” angles) as there were no differences in the BOLD response for this variable.

**Figure 8:**
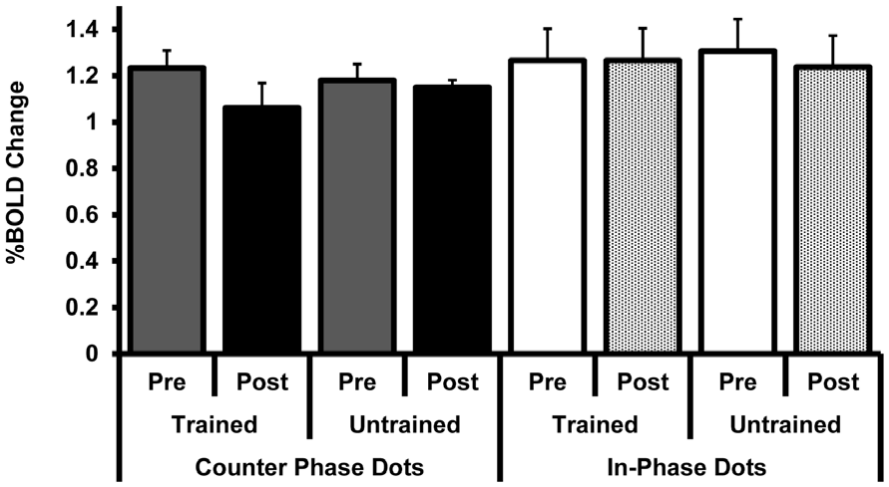
The response of V1 (%BOLD change) pre and post training with counter-phase dots (filled bars) or in-phase dots (open/textured bars). There were no reliable training-related changes in activation. Post training data are collapsed across angle size (“same” and “smaller” angles) as there were no differences in the BOLD response for this variable.

The post-training BOLD response at hMT+ and V1 did not differ between the same and smaller angle stimuli for any condition (MT; counter-phase trained axis t_8_<1, counter-phase untrained axis t_8_ = <1, in-phase trained axis t_6_<1, in-phase untrained axis t_6_ = <1, V1; counter-phase trained axis t_8_ = −1.9, p = 0.1, counter-phase untrained axis t_8_ = −1.1, p = 0.3, in-phase trained axis t_6_<1, in-phase untrained axis t_6_ = <1). We therefore collapsed across this variable for subsequent analyses to facilitate the comparison between the pre and post training data (Figure 7 for hMT+ and Figure 8 for V1).

A mixed ANOVA with factors of area (hMT+ vs. V1), motion axis (trained vs. untrained), training (pre vs. post training) and dot phase (counter-phase vs. in-phase) revealed a significant interaction between area, motion axis, and training (F_1,14_ = 4.7, p = 0.05). No other main effects or interactions were significant. To further investigate the relationship between motion axis and training we conducted a series of repeated measures ANOVAs on the BOLD response data for each area (hMT+ and V1) for each dot phase group (counter-phase and in-phase). For the participants trained on counter-phase dots, there was a significant interaction between motion axis and training for hMT+ (F_1,8_ = 5.3, p = 0.05) and a marginal main effect of training (F_1,8_ = 3.9, p = 0.08). Post hoc t-tests revealed a significant reduction in BOLD response for the trained motion axis from pre training to post training (t_8_ = 2.7, p = 0.03) that was not present for the untrained motion axis (t_8_ = 1.3, p = 0.2). No significant interactions or main effects were present at V1 for the group trained on counter-phase dots (p>0.4), or at either hMT+ or V1 for the group trained on in-phase dots (p>0.4).

**Figure 9:**
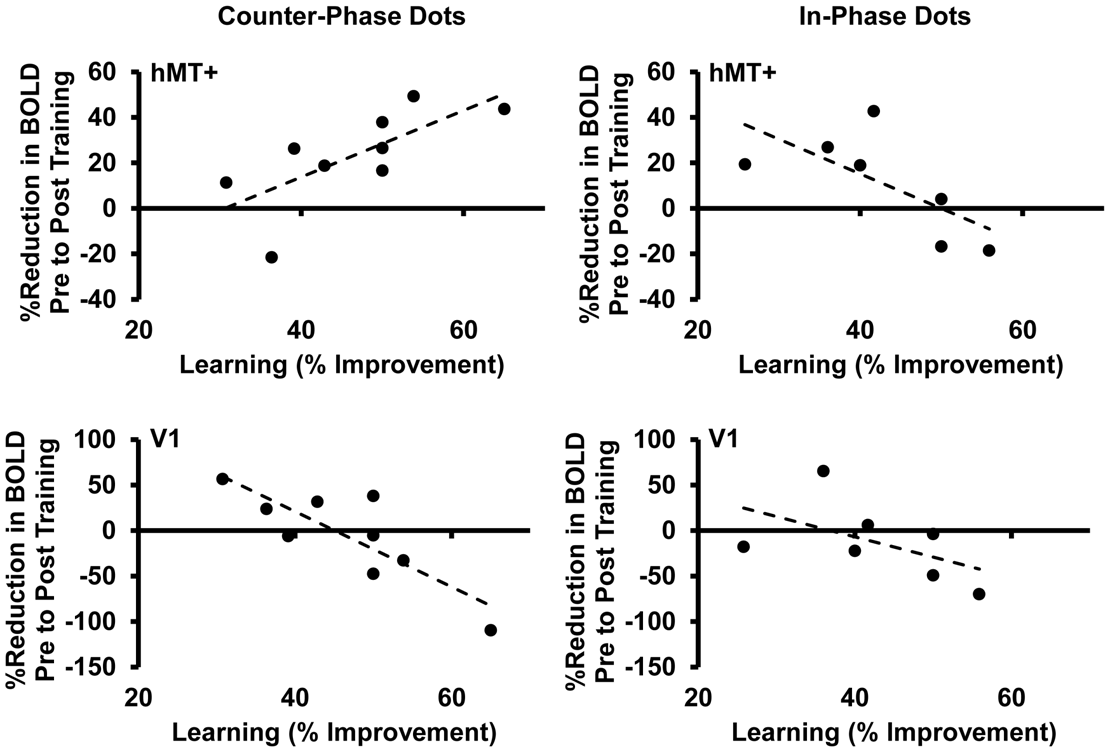
The relationship between the change in BOLD response and the amount of learning. The percent reduction in BOLD response pre to post training for the trained motion axis is shown on the Y axis and the percent improvement in 75% correct threshold a result of learning is shown on the X axis. Each data point represents an individual participant. Data are shown for hMT+ (top row) and V1 (bottom row) for counter-phase dots (left column) and in-phase dots (right column). Post training data are collapsed across angle size (“same and “smaller” angles).

Although variability in the amount of learning was constrained by our training protocol, there was evidence for a relationship between the BOLD response at hMT+ and the amount of learning that took place for the participants trained on counter-phase dots. Specifically, for this group, greater amounts of learning (the percent reduction in angular difference achieved during training) were associated with a greater reduction in BOLD response at hMT+ from pre to post training (Pearson’s R = 0.7, p = 0.03, Figure 9). The opposite relationship was found at V1 (R = −0.8, p = 0.006). For the group trained on in-phase dots, greater learning was associated with less reduction, and in some cases an increase, in BOLD response from pre to post learning at both hMT+ (Pearson’s R = −0.7, p = 0.09) and V1 (R = −0.5, p = 0.2).

A bootstrap analysis (10,000 repetitions with replacement) was conducted to assess whether the relationships between training related changes in BOLD and behaviour (Figure 9) were reliably different for each area (hMT+ and V1) and for each group (counter-phase vs. in-phase). Pearson’s R was calculated for each of the two conditions being compared and the difference between these two R values was computed. The 10,000 differences were then ranked from smallest to largest. The 250^th^ and 9750^th^ values in the ranked list were taken to represent the lower and upper limits of the 95% confidence interval of the difference in R between the two conditions. The range of these confidence intervals did not include zero for the counter-phase dot group hMT+ vs. V1 (1.8 to 6.4), the counter-phase dot group hMT+ vs. the in-phase dot group hMT+ (0.8 to 4.7) and the counter-phase dot group hMT+ vs. the in-phase dot group V1 (0.05 to 7.7). All other comparisons generated confidence intervals that included zero. No systematic relationships between the BOLD response and behaviour were found for the untrained motion axis orientation. In addition, an exploratory, whole brain, voxel-wise, random effects ANOVA analysis did not reveal any training related changes in the activation of visual brain areas for the group trained on in-phase dots.

In summary, training on counter-phase dots reduced the response of hMT+ (Figure 7A), and greater amounts of learning were associated with a larger reduction in BOLD response at hMT+ after training (Figure 9).

#### Post training motion opponency

The data from our primary experiment indicated that training with counter-phase dots reduced the BOLD response at hMT+ and that a greater reduction was associated with a greater amount of learning. To investigate the possibility that any training-induced effects on hMT+ activation were due to a change in motion opponency, we compared, in a parallel experiment, the relative response of hMT+ to counter-phase and in-phase dot stimuli before and after counter-phase dot training. We reasoned that if the reduction in hMT+ BOLD response for counter-phase dots relative to in-phase dots became more pronounced after training, this would be consistent with an increase in suppression at hMT+. These measurements were made on a group of 8 participants who were trained with the counter-phase dots and scanned with both counter-phase and in-phase dots pre and post training (Table 1). All stimuli were presented at the participant-specific angular difference that gave rise to 75% correct (equivalent to the “smaller angle” at post-training in the primary experiment).

**Figure 10:**
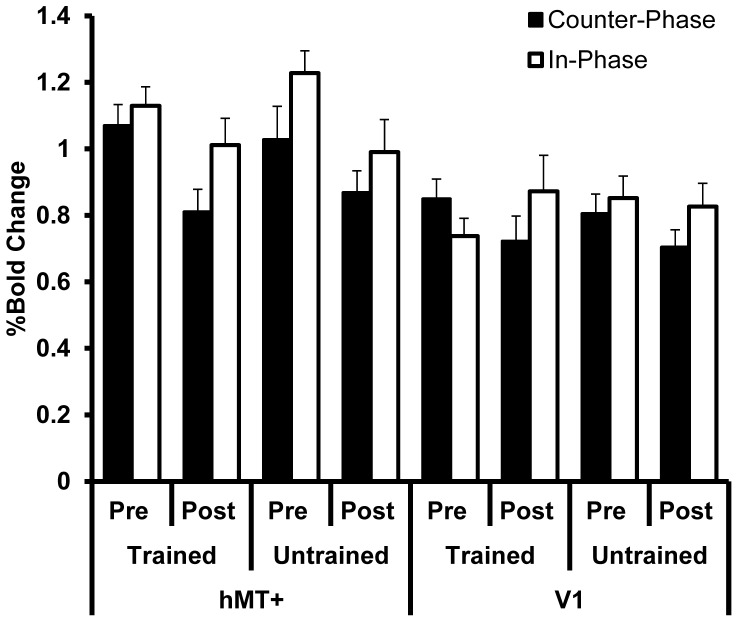
The effect of counter-phase dot training on the response of hMT+ and V1 to counter-phase and in-phase dot stimuli. Filled bars indicate counter-phase dots and open bars in-phase dots. Training increased the difference between in-phase and counter-phase dot responses at hMT+ for the trained motion axis.

**Figure 11:**
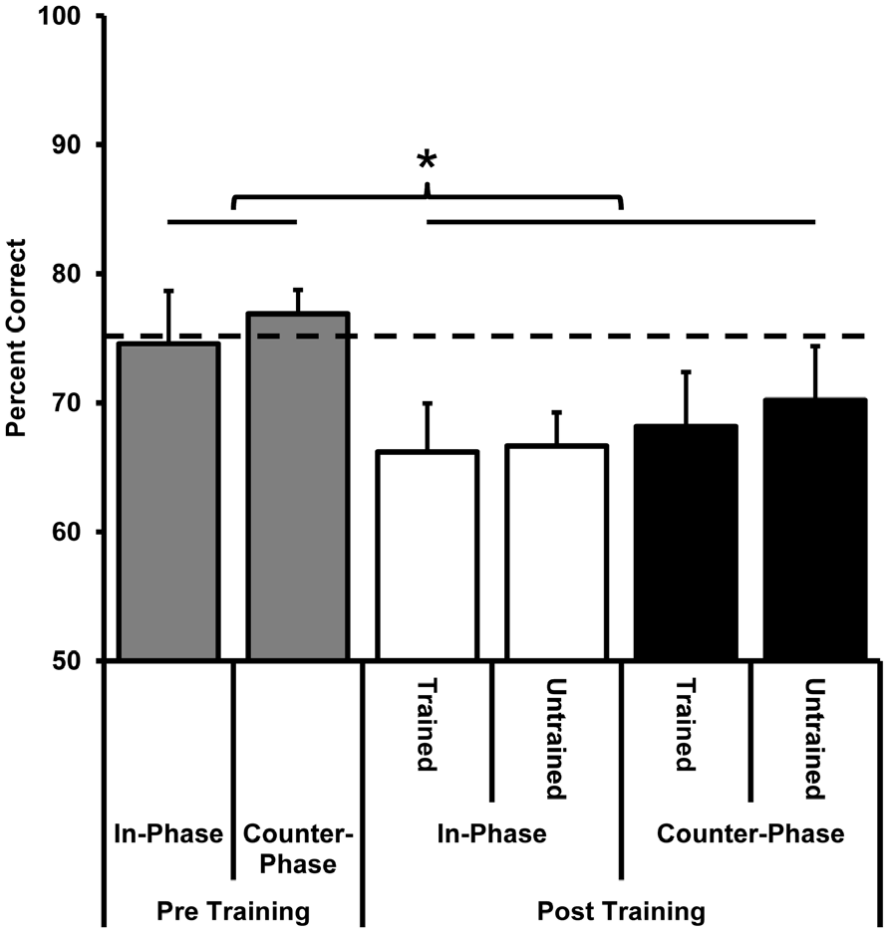
Behavioural accuracy during scanning for participants trained on counter-phase dots and scanned post training with both counter-phase and in-phase dot stimuli (n = 7). Pre training data are collapsed across motion axis orientation. The dashed line indicates 75% correct accuracy measured outside of the magnet. Behavioural accuracy during scanning did not differ between the two dot phase conditions (p>0.05) or between the trained or untrained motion axis (p>0.05), however post training behavioural accuracy during scanning was lower than pre training performance, F_1,7_ = 6.4, p = 0.04.

Training increased the difference in the BOLD response of hMT+ to in-phase vs. counter-phase dots for the trained motion axis (Figure 10, compare the difference between the two left-most pairs of black and white bars). In particular, while the post training responses were lower for both conditions, the reduction in response was more pronounced for the counter-phase dots than the in-phase dots. To quantify this effect, the BOLD response (%BOLD change) for the counter-phase dots was subtracted from the BOLD response for the in-phase dots for each condition. This calculation was performed separately for each participant and provided an estimate of the suppressive effect of the counter-phase dot stimulus relative to the in-phase dot stimulus. A within-subjects ANOVA conducted on the difference scores with factors of area (hMT+ vs. V1), motion axis orientation (trained vs. untrained), and time (pre-training vs. post-training) revealed a significant interaction between motion axis and time (F_1,7_ = 7.1, p = 0.03). This interaction was due to larger difference scores for the trained motion axis relative to the untrained motion axis at both hMT+ and V1 post training. When the data for hMT+ and V1 were analysed separately it was apparent that the interaction effect was significant within hMT+ (F_1,7_ = 14.7, p = 0.006) but not within V1 (F_1,7_ = 3.3, p = 0.11). On average, the difference in BOLD response at hMT+ for in-phase vs. counter-phase dot stimuli increased by 75%±89% (t_7_ = 2.4, p = 0.05) for the trained motion axis, which is consistent with a training related increase in motion opponency at hMT+. This result could not be accounted for by differences in task difficulty between the two conditions (Figure 11) as behavioural accuracy was equivalent between the two dot motion conditions pre training (compare the grey bars in Figure 11) and post training (compare the open and closed bars in Figure 11). It is apparent, however, that the difference in BOLD response to counter-phase dots vs. in-phase dots was smaller for the trained axis than the untrained axis pre-training (Figure 10), which contributes to the interaction effect we report. We randomized training angles across participants and collected data in common scanning runs using randomly sequenced blocks to control for any measurement bias. In addition training angles were assigned after the pre-training fMRI data were collected but before the fMRI data had been analysed to avoid any subject or experimenter bias. Given the relatively small number of participants, the observed pre-training difference is likely due to anisotropy in orientation discrimination performance for some of the participants. We statistically controlled for this idiosyncrasy by assessing the training effects blocked by orientation. In the context of this pre-training difference, it is still the case that training increased the difference in BOLD response between counter-phase and in-phase dots in the trained orientation and not in the untrained orientation.

We did not find evidence for increased motion opponency for the untrained motion-axis even though there was a significant transfer of learning (t_7_ = 2.6, p = 0.04). This may have been due to the smaller extent of learning that occurred for the untrained motion-axis. For example, the two participants with the largest amounts of transfer, comparable in magnitude to the amount of learning found for the trained orientation, showed evidence for an increase in motion opponency at hMT+ for the untrained motion-axis (65% and 44% behavioural transfer and 23% and 112% increase in the difference in BOLD response to in-phase vs. counter-phase dots, respectively).

## Discussion

The aim of this study was to investigate the mechanisms involved in the learning of a motion task while MT was suppressed via motion opponency. In particular, we investigated whether perceptual learning would reduce motion opponency within MT or whether a lower level area, in this case V1, would become responsible for task learning. Contrary to expectation, our data did not support either of these possibilities, but demonstrated that perceptual learning with a motion-opponent stimulus reduced MT activation. This reduction in hMT+ activation was significantly stronger for the trained than the untrained motion axis and greater amounts of learning (in terms of behavioral performance) were correlated with a greater reduction in BOLD response. This relationship was specific to area hMT+ for the group trained on counter-phase dots (which suppress hMT+), supporting the idea that the reduction in BOLD was linked to learning. Training with in-phase dots (which do not suppress hMT+) also appeared to reduce activation at hMT+; however this effect was not statistically significant and was not specific to the trained motion axis. V1 activity was stable pre and post training, particularly for the group trained on in-phase dots. In an additional experiment we found that training on counter-phase dots increased the difference in the response of hMT+ to counter-phase vs. in-phase dots, possibly due to an increase in motion opponency within hMT+ for this particular stimuli.

It is notable that training with counter-phase dots also resulted in a trend for reduced activation at hMT+ when participants viewed stimuli presented along the untrained motion axis. This may have been due to the significant behavioural transfer of learning from the trained to the untrained motion axis that we observed. In previous studies using a different training method we found little transfer to untrained motion-axes [35], [41]. Here, using the method of constant performance, we found reliable behavioural transfer to an untrained stimulus. This interesting and unexpected result is beyond the scope of this study, but deserves further investigation.

A central premise of this study, and the prior psychophysical work on which it was based [35], [41], was that counter-phase dot motion would suppress MT activity via motion opponency. This premise was based on previous neurophysiological [36]–[38] and brain imaging work [38], [39] demonstrating a weaker net response from MT for stimuli with locally balanced motion signals. The pre-training data collected as part of the present study confirmed that our counter-phase dot stimuli induced less activity in hMT+ than the in-phase dot stimuli, even though behavioural task performance did not differ between the two conditions. This effect was not large, but it was statistically reliable and of a comparable magnitude to previous reports [38]. V1 did not show a differential response to counter-phase vs. in-phase dots, further supporting the argument that the difference found at hMT+ was due to motion opponency, a phenomenon that is mostly absent at V1 [36].

The reduction in BOLD response at hMT+ induced by counter-phase dot training we report for the trained motion axis was revealed by comparing pre and post training data. Although comparisons between scanning sessions can be prone to baseline shifts, the fact that this reduction was significantly greater for the trained than the untrained motion axis indicates that the effect was not simply due to inter-scan variability. In addition, only small reductions were found at hMT+ for the group trained on in-phase dots and V1 activity was remarkably stable across the two scanning sessions.

One possible explanation for the change in hMT+ BOLD response we observed after counter-phase dot training involves noise reduction. It has been shown that the average response of cells within MT to counter-phase dot motion is equivalent to non-directional flicker noise [36]. This suggests that, on average, MT neurons may not have carried useful task-relevant signals when participants observed the counter-phase dot stimuli. Therefore training on counter-phase stimuli may have resulted in a further suppression of MT responses in order to reduce noise. This explanation is supported by the finding that training on counter-phase dots and testing with both counter and in-phase dots pre and post training revealed a training-related reduction in hMT+ activity that was selective for counter-phase dots. It is possible that this effect was due to a learning-induced increase in the strength of motion opponent interactions within hMT+, which suppressed the uninformative response of this area to the counter-phase dot stimulus.

Based on our current data, we cannot determine whether the reduced response of hMT+ reflected a general attenuation of the whole area or a specific reduction in the activity of cells with strong motion opponent responses, leaving non-opponent motion cells available for task performance. In general terms, one may speculate that an overall attenuation of MT would have resulted in learning-related changes at V1. This is because V1 contains motion sensitive cells that would not have been suppressed by the counter-phase dot stimulus [36] and therefore could have contributed to task performance. As no reliable changes in the response of V1 were observed in the current study, it is possible that a sub-set of cells within hMT+ may have mediated task performance [47].

Prior neuroimaging studies have also reported a reduction in the BOLD response within regions of the visual cortex as a result of perceptual learning [10], [11], [48]. However this is not a ubiquitous finding, as a number of studies have reported significant increases in BOLD response within the early visual areas after learning [8], [12], [13], [15], [49]. It is likely that this variation between studies is due to differences in the types of task used for training and the training regimes employed [49].

One unexpected result of this study was that despite pronounced improvements in task performance, no statistically reliable changes in visual cortex activation were found for the group of participants trained on in-phase dots. This was the case both for a region of interest analysis targeting V1 and hMT+ and a voxel-wise analysis designed to identify training-related changes in activation anywhere within the visual cortex. These results are in agreement with the neurophysiological data reported by Law and Gold [26] which demonstrated that learning of a motion coherence task did not induce long lasting changes in MT but modified the read out of signals from MT by higher level area LIP. Theories of perceptual learning relying primarily on changes in the read out of brain areas that receive inputs from early regions of the visual cortex can account for wide range of behavioural effects associated with perceptual learning, including stimulus specificity [50]. It is certainly possible that training with in-phase dots resulted in a reweighting of motion direction signals generated by units within V1 and MT at a decision stage, perhaps occurring within LIP. However, we cannot rule out the possibility that training with in-phase dots altered the response of MT (or V1) in a way that is not reliably reflected in the magnitude of the BOLD response. For example, the use of multi-voxel pattern classification analysis has shown that perceptual learning can improve the ability of areas such as V3 and V3A to represent differences between trained stimuli [51], [52]. Such changes may not be reflected in the overall BOLD response of an area. Furthermore, changes in the BOLD response of regions within the visual cortex may occur early in learning and return to baseline once learning has reached asymptote levels [15]. As we only collected fMRI data at the start and end of training, it is conceivable that changes at MT may have occurred early during training for the in-phase dot group and returned to baseline prior to the post-training scanning session.

In conclusion, training with suppressed MT resulted in a further attenuation of hMT+ activity. Furthermore, greater reductions in the response of hMT+ were correlated with greater amounts of learning. The relationship between a reduced BOLD response at hMT+ and greater learning was specific to training with suppressed MT and may reflect a reduction of noise in the neural representation of the training stimulus. These results suggest that the effect of perceptual learning on early visual areas may critically depend on the specific stimulus presented during training and that, in certain situations, perceptual learning of a motion task can reduce the response of MT.
